# Predictors of Condom-use among Young Never-married Males in Nigeria

**DOI:** 10.3329/jhpn.v29i3.7875

**Published:** 2011-06

**Authors:** Kolawole Azeez Oyediran, Oluwadaisi Isaac Feyisetan, Toyin Akpan

**Affiliations:** ^1^MEASURE Evaluation/JSI, 90 Nelson Mandela Street, Asokoro, Abuja, Nigeria; ^2^Northern Education Initiative, Block 18, Ministry of Education, Shehu Kangiwa Secretariat, Sokoto, Sokoto State, Nigeria; ^3^AURICLE Services Limited,37A Ladipo Kasumu Street, By Bisi Ogabi Street, Ikeja, Lagos, Nigeria

**Keywords:** Acquired immunodeficiency syndrome, Condom-use, HIV, Sex behaviour, Sexually transmitted infections, Youth, Nigeria

## Abstract

This study examined the factors that influence condom-use among young never-married males in Nigeria. Such information can help improve the design of a prevention programme for young never-married, especially, males to reduce their vulnerability to sexually transmitted infections (STIs) and HIV/AIDS transmission. Data were derived from the 2003 Nigeria Demographic and Health Survey (NDHS). Analysis of data was restricted to 827 males aged 15-24 years, who had never married or lived together with a woman. Both descriptive and analytical methods were used for assessing the net effects of socioeconomic factors on condom-use. The analysis used logistic regression models for determining the predictors of sexual behaviour and condom-use among young never-married males in Nigeria. About 43% of the study population was sexually experienced, and the use of a condom remained low. One in five reported the use of a condom at sexual debut. Level of education, place of residence in childhood, urban/rural region, religious affiliation, economic status index, and exposure to mass media were associated with sexual experience and use of protective measures. Economic status index and mass-media exposure were associated with the use of a condom by the respondents during their last sex encounter. About two-fifths (43%) of the young unmarried Nigerian men were sexually experienced but the condom-use remained low, thus making this sub-group of Nigerian population highly vulnerable to STIs, including HIV/AIDS.

## INTRODUCTION

Adolescence is a transition period between childhood and adulthood. This is a time when many young people experience critical life-defining changes, such as their first sexual experience, marriage, pregnancy, and parenthood. Initiation of sexual intercourse has been described as a milestone in the physical and psychological development of men and women in all societies (1). This episode in life is taking place in a different socioeconomic context from previous generations. Results of studies revealed that the circumstances of the first sexual encounters of adolescents have immediate and longer-term consequences for their sexual health later in life (2-3). Some instances of very early sexual intercourse have been reported to be involuntary—for example, a young person being raped or a victim of incest or turned to prostitution because of financial need (1,4-5). A study in Ibadan, Nigeria, reported that 8% and 5% of in-school adolescents and adolescent apprentices respectively in Ibadan reported to have been raped (6). Results of another study in Plateau state showed that 11% of secondary school students aged 12-21 years had had forced sexual intercourse (7).

Sexual behaviour of adolescents is important not only because of the possible reproductive outcomes but also because risky sexual behaviours, such as unprotected sex and low and inconsistent use of a condom during sexual intercourse, have been associated with HIV infection. Early sexual initiation poses health risks for both young men and women. Most young adults who entered into a sexual relationship for the first time did not use any form of contraception and were ignorant of the consequences of their acts (8-10) leaving them vulnerable to unintended pregnancies and unplanned parenthood. Results of a study of adolescent sexuality in Peru showed that 38% of male youths used condom at the time of first intercourse (8). In Ibadan, 43.9% of in-school adolescents with single parents did not know that pregnancy could result from first coitus (9). The visible consequences of teenage sexual behaviour in Nigeria are high rates of out-of-wedlock pregnancies among adolescents, abortions, and sexually transmitted infections (STIs) which increase the risk of HIV infections (11-15). For example, 42% of adolescent girls in a rural community in Rivers state had had induced abortion or STIs, including gonorrhoea (12). In Jos (Plateau state), 24% of patients who attended STI clinics were aged less than 25 years (14). In Calabar (Cross River state), 72% of patients admitted to hospitals for complications of abortion were aged 12-20 years (11).

In Nigeria, sexual activity among adolescents is a concern of public-health priority for government officials, donors, and programme managers because of the serious outcomes usually accompanying teenage unprotected sexual exploitation (16-18). This concern is reflected in the growing number of reproductive health interventions aimed at young never-married males that have been implemented in many settings in recent years and increasing level of resources earmarked for such programmes. Results of studies on adolescent sexuality suggest that young Nigerians are becoming sexually active at an earlier age (9,19-22). According to the 2003 Nigeria Demographic and Health Survey (NDHS), 75.5% of women aged 25-49 years had sexual intercourse by the age of 20 years, and 39.3% of men aged 25-59 had sexual intercourse by the age of 20 years. In a related analysis of the 1999 NDHS, 31.5% of unmarried female youths aged 15-24 years were sexually experienced, and the median age of sexual debut was 16.6 years (22). Only one-fifth of these sexually-active unmarried female youths, however, used a condom at the last sexual intercourse, hence making them vulnerable to unplanned parenthood and STIs, including HIV.

Sensitization and information programmes on the consequences of unprotected sexual intercourse, including HIV/AIDS, have been implemented in Nigeria, along with efforts to promote the use of condoms. These programmes have, however, had limited impact in the translation of knowledge about HIV/AIDS and prevention methods into the adoption of healthy sexual behaviour (23). Evidence also suggests that adolescents have sex because of peer pressure, curiosity, love, promise of marriage, and to have fun (3,24-25). Given that parents of adolescents do not discuss sexual matters with their offspring, information about sex is largely obtained from peers and garbled, inaccurate literature. Moreover, information provided by mass media is often distorted, and peers tend to be misinformed as they lack adequate and correct knowledge of sexual and reproductive health. Most adolescents, especially males in Nigeria, do not use pregnancy-prevention methods, particularly condom, for a number of reasons. The reasons include misconception that their female partners will not get pregnant; the unavailability of youth-friendly clinics from which they can seek advice on contraceptive methods; and refusal to attend clinics for reproductive health issues due to lack of privacy.

Designing an effective programme to promote res-ponsible reproductive health behaviour among young adults in Nigeria requires a better understanding of the factors relating to their sexual behaviours. Although scholars have documented factors associa-ted with reproductive health behaviour among Nigerian youths, a few of these have examined sexual behaviour of young never-married males and in particular condom-use at sexual debut. This study examined the predictors of condom-use at sexual debut among young never-married males in Nigeria. Condom-use during the first sexual experience warrants special attention in light of the spread of STIs and the HIV/AIDS pandemic and the increase in unwanted and unplanned pregnancies that may lead to discontinuation of schooling. Overall, this paper focused on understanding the factors affecting condom-use by young never-married males at both sexual initiation and the last sexual act.

## MATERIALS AND METHODS

The study used data from the 2003 NDHS. This nationally-representative sample survey covered 7,864 households located in both urban and rural settings. The area-sampling frame for the survey was based on the enumeration area-maps prepared by the National Population Commission (NPC) for conducting the 1991 census. Selection of sample was made in two stages: first, 365 clusters were selected with equal probability. Second, within each of these 365 clusters, a complete listing was done of all residents in sampled households, from which male and female respondents were interviewed. Details of the research design are available in the 2003 NDHS report (26). In total, 7,327 (95%) of the 7,684 households selected for the study were found. The shortfall was largely due to vacant dwelling structures found at the time of survey. Of the 7,327 existing households, 7,225 (99%) were successfully interviewed. In these households, 7,985 women and 2,572 men were eligible for the individual interviews. However, 7,620 women aged 15-49 years and 2,346 men aged 15-59 years were successfully interviewed, representing a response rate of 95% and 91% for women and men respectively. Of the male respondents, 827 (35%) were aged 15-24 years and had never married or lived together with a woman, and 355 had had sexual experience. Analysis here is restricted to this sub-population.

Analysis focused on four reproductive health behaviours: whether respondents had had sex; whether they had sex in the four weeks before the interview; whether they had used condom at first sexual intercourse and whether condom was used at the last sexual intercourse. Their demographic characteristics were included in the analysis to identify factors for possible intervention and to act as control variables in the analytical models. The characteristics considered were age, the highest level of education completed, place and region of residence, religious affiliation, media exposure, and wealth quintile. Analysis of the first reproductive health behaviour—whether the respondents had had sexual intercourse—was based upon responses provided by all 827 sample respondents. However, analysis of the remaining three reproductive health behaviours was based on 355 respondents who had had sexual intercourse.

Both descriptive and analytical analyses were conducted to ascertain the association and net effect of the background characteristics of the respondents on reproductive health behaviours—sexual activity and condom-use—when controls for the selected background characteristics were introduced. The statistical analyses were undertaken in two phases. Bivariate relationships between each of the selected characteristics/predictors and reproductive health behaviours of interest were established. The statistical significance of difference among categories of discrete reproductive health behaviours was tested through Pearson's chi-square statistic.

Background characteristics for which statistically significant bivariate associations were observed were retained for subsequent multivariate analyses. The four reproductive health behaviours of interest (ever had sex, condom-use at first sex, condom-use at last sex, sexually active in the previous four weeks) were binary outcomes. Logistic regression was used for determining the effects of predictor variables on selected reproductive health behaviours. The logistic regression model also offers ease of interpretation through the use of odds ratios. The logistic regression function has the form In (p/q)=B_0_+B_1_X_1_+ … +B_k_X_k_, where p is the probabili-ty that a respondent used a condom during sexual debut or the last sexual act; q (or 1-p) is the probability that the respondent did not use a condom at sexual debut or the last sexual act. B_0_, B_1_, … B_k_ are regression coefficients, and X_1_, X_2_, … X_k_ are factors. The exponential of the regression coefficients of the parameter estimated gives the odds ratios in the logistic regression models, which is interpreted as the likelihood of condom used under the given scenario

## RESULTS

### Profile of respondents

In total, 827 young never-married males, aged 15-24 years, who were interviewed during the 2003 NDHS, were analyzed in the study. Table 1 presents the basic demographic characteristics of the study population. About 54% of them were aged 15-19 years while the remaining 46% were aged 20-24 years. Their median age was 19 years. The survey indicated that more than half of the respondents resided in rural areas, and just little over 47% had place of residence during childhood in the countryside. Spatial distribution of the study population showed that 52% of them resided in the north while the remaining 48% resided in the three regions in the southern part of the country.

**Table 1.** Percentage distribution of young never-married males aged 15-24 years by selected sociodemographic characteristics

Characteristics	No. (n=827)	%
Age-group (years)		
15-19	448	54.2
20-24	379	45.8 19.0*
Place of residence		
Urban	387	46.8
Rural	440	53.2
Region of residence		
North-central	160	19.3
Northeast	107	12.3
Northwest	169	20.4
Southeast	113	13.7
South-South	127	15.4
Southwest	151	18.3
Childhood place of residence		
City	174	21.1
Town	262	31.7
Countryside	390	47.2
Educational level		
No education	71	8.6
Primary	174	21.0
Secondary	528	63.8
Tertiary	54	6.5
Religious affiliation		
Catholic	167	20.2
Protestant	124	15.0
Other Christians	188	22.7
Muslim	341	41.2
Traditional/others	7	0.8
Wealth quintile		
First	140	16.9
Second	124	15.0
Third	152	18.4
Fourth	213	25.8
Fifth	198	23.9
Frequency of reading newspapers or magazines		
Never	392	47.5
Less than once a week	216	26.2
At least once a week	158	19.1
Almost every day	60	7.3
Frequency of watching television		
Never	190	23.0
Less than once a week	193	23.3
At least once a week	196	23.7
Almost every day	247	29.9
Frequency of listening to radio		
Never	45	5.4
Less than once a week	104	12.6
At least once a week	192	23.2
Almost every day	484	58.5

Source of data: Nigeria demographic and health survey 2003 (24).

* Median age of respondents

About 70% of the study men had some secondary education. Only 6% of them had post-secondary education, and 8.6% had no formal education. Christians constituted a majority (58%) of the sampled respondents, with three in five young never-married males being Christians. Muslims constituted 41.2%, and less than 1% was adherent to indigenous religions. Another important background characteristic considered in the analysis was the wealth quintile. The wealth quintile constructed from the household facilities was used as a proxy for economic status. The level of wealth quintile ranged from the first to the fifth quintile, corresponding to the least and most well-off res-pectively. The wealth quintile revealed that about one in four young never-married males was from households classified in the fifth quintile.

Table 1 reveals a high level of exposure to electronic mass media. Most (94%) respondents listened toradio at least once in a while, and 59% of the young never-married males reported listening to radio every day. About a quarter (23%) had never watched television, and about 30% watched television every day. The results indicate widespread access of the participants to media.

### Sexual behaviour

Overall, about 43% of the respondents had ever had sexual intercourse, and the median age at sexual debut was 18 years. About one-fifth of the study population had sexual relations in the month and of those who were sexually experienced, about 47 had sexual relations in the four weeks before interview. Table 2 shows the distribution of sexually-experienced young never-married males and current sexual activity by selected sociodemographic factors.

**Table 2.** Percentage distribution of young never-married males, aged 15-24 years, who were sexually experienced and current sexual activity among sexually experienced by selected characteristics

Characteristics/variable	Ever had sex (n= 827)		Sexually active in the last4 weeks among thesexually experienced (n=355)	
	No.	%	No	%
Age-group (years)				
15-19	448	24.8	126	44.9
20-24	379	62.1	283	47.2
Place of residence				
Urban	387	39.6	179	33.9
Rural	440	45.1	230	53.8
Region of residence				
North-central	160	52.1	86	50.9
Northeast	107	47.3	59	58.5
Northwest	169	25.0	54	61.4
Southeast	113	43.9	56	17.0
South-South	127	48.0	71	51.6
Southwest	151	52.2	83	27.9
Childhood place of residence				
City	174	35.4	66	34.7
Town	262	45.5	137	45.2
Countryside	390	44.0	205	50.9
Educational level				
No education	71	31.6	34	57.2
Primary	174	39.6	90	51.1
Secondary	528	45.1	256	45.4
Tertiary	54	56.5	29	29.8
Religious affiliation				
Catholic	167	47.7	88	40.1
Protestant	124	51.5	64	52.8
Other Christians	188	46.3	100	32.4
Muslim	341	37.0	153	56.5
Traditional/others	7	56.7	4	27.2
Wealth quintile				
First	140	50.5	84	60.7
Second	124	35.1	55	57.5
Third	152	40.6	71	53.4
Fourth	213	38.7	89	42.9
Fifth	198	49.1	110	26.6
Frequency of reading newspapers or magazines				
Never	392	34.2	178	53.8
Less than once a week	216	51.5	111	42.9
At least once a week	158	49.8	84	46.7
Almost every day	60	61.7	35	25.3
Frequency of watching television				
Never	190	36.9	87	63.8
Less than once a week	193	47.3	103	50.7
At least once a week	196	40.5	90	55.0
Almost every day	247	45.5	128	27.6
Frequency of listening to radio				
Never	45	23.7	18	64.1
Less than once a week	104	25.5	36	49.4
At least once a week	192	36.0	83	51.6
Almost every day	484	50.9	271	43.6

Source of data: Nigeria demographic and health survey 2003 (24)

Results of analysis revealed that the proportions of the respondents who were sexually experienced and those who had sexual intercourse in the previous four weeks before the interview survey increased with age. For instance, one-fourth of the young never-married male respondents aged 15-19 years and 62% of their older peers aged 20-24 years had ever had sex. The corresponding figures with respect to sexual activity in the past month were about 45% and 47% respectively. Table 2 further reveals that the young never-married males from the southeast commenced sexual activity about six months earlier than their counterparts from the northern and southwestern regions.

Rural residents tended to initiate sexual relations earlier than did urban residents (about a one-year difference) and were slightly more sexually experienced and more sexually active in the last four weeks before the interview. For instance, 45% of the young never-married rural males were sexually experienced compared to 39.6% of their urban counterparts. Childhood place of residence somewhat supported the notion of less-frequent sexual activity among those who had some urban exposure. However, the age at sexual debut was generally higher among those with higher level of education compared to those with lower level of education. This finding indicates that young never-married males with no formal education started sexual activity two years earlier than those with tertiary education.

Religion is believed to have a profound impact on individual behaviour and views (27,28). Even in the face of modernization and its consequences on cultural practices, religion seems to still shape beliefs about sexual activity. Therefore, young never-married males who were more religious were more likely to hold types of beliefs that may discourage sexual activity. The relationship between religious affiliation and reproductive health behaviour, particularly sexuality, is likely to be mediated by social and demographic factors, such as education of the respondent. Table 2 shows that young never-married Muslim males were less sexually experienced but more sexually active in the month preceeding the survey than their Christian counterparts: about 37% of young never-married Muslim males in Nigeriareported being sexually experienced compared to 48% of their counterparts who were Catholic. In addition, Muslim respondents started sexual activity about one year later than Christian respondents and two years later than those adherent to other faiths. A higher proportion of young Muslim men reported being more sexually active in the last four weeks than their Christian counterparts (57% vs 32%).

Table 2 further shows that the wealth quintiles were inversely associated with being sexually active in the month before the survey. First, those from the first and the fourth wealth quintile started sexual relations one year earlier than those from other wealth quintiles. Level of access to media information was positively related with the level of sexual activity, although the median age at initiation of sex was inversely related with access to information. Respondents with low access to media information started sexual activity almost 12 months earlier than those with better access to information.

In the logistic regression model (Table 3), young never-married males aged 20-24 years were (5.49 times) more likely than those aged 15-19 years to have had sex. The odds of engaging in premarital sex were 1.64 times higher among young never-married rural males than their urban counterparts, which is consistent with the findings of earlier studies in Nigeria (20,22). Region of residence was also associated with engaging in premarital sex: compared to young never-married males in the north-central region, those in the northeast, northwest, and southeast regions were significantly less likely to engage in premarital sex. Those in the northeast were about half as likely to engage in premarital sex compared to those in the north-central states. Young never-married males from households classified into second quintile and fourth quintile were less likely to have premarital sex compared to those from households ranking in the first quintile.

**Table 3.** Logit regression results for correlates of young never-married males (aged 15-24 years)—reports of whether sexually experienced and current sexual activity among the sexually experienced, Nigeria, 2003

Characteristics	Sexually experienced (n=355)		Sexually active in the last four weeks (n=355)	
	Odds ratio	Standard error	Odds ratio	Standard error
Age-group (years)				
15-19	1.00		1.00	
20-24	5.49**	1.04	1.45	0.42
Place of residence				
Urban	1.00		1.00	
Rural	1.64*	0.42	1.49	0.55
Region of residence				
North-central	1.00		1.00	
Northeast	0.50*	0.18	0.78	0.35
Northwest	0.14**	0.05	0.83	0.44
Southeast	0.46*	0.15	0.24**	0.12
South-South	0.75	0.25	1.54	0.63
Southwest	0.78	0.25	0.67	0.27
Educational level				
No education	1.00		1.00	
Primary	1.05	0.41	2.05	1.06
Secondary	0.64	0.26	2.32	1.31
Post-secondary	0.53	0.31	2.39	1.92
Religious affiliation				
Catholic	1.07	0.36	0.51	0.26
Protestant	0.95	0.30	0.77	0.37
Other Christians	0.77	0.23	0.29*	0.16
Muslim	1.00		1.00	
Wealth quintile				
First	1.00		1.00	
Second	0.55*	0.15	0.89	0.37
Third	0.62	0.21	1.19	0.60
Fourth	0.55*	0.18	0.73	0.34
Fifth	0.85	0.32	0.93	0.56
Frequency of reading newspapers or magazines				
Not at all	0.43	0.22	1.61	0.98
Less than once a week	0.86	0.43	1.13	0.72
At least once a week	0.95	0.46	2.04	1.24
Almost every day	1.00		1.00	
Frequency of watching television				
Not at all	1.40	0.53	3.10*	1.68
Less than once a week	1.87*	0.58	1.67	0.85
At least once a week	1.28	0.34	1.97	0.77
Almost every day	1.00		1.00	
Frequency of listening to radio				
Not at all	0.24*	0.16	1.38	0.95
Less than once a week	0.35**	0.13	0.75	0.44
At least once a week	0.66	0.18	1.10	0.36
Almost every day	1.00		1.00	

*p<0.05;

**p<0.01;

Source of data: Nigeria demographic and health survey 2003 (24).

1.00=Reference category

With respect to correlates of current sexual activity in the last four weeks preceding the survey, loresidence, religious affiliation, and frequency of watching television were significantly associated with whether the young never-married males engaged in premarital sex in the last four weeks before the survey. Those who resided in the southeast region were almost four times less likely to engage in sex in the previous four weeks compared to their counterparts from the north-central region.

### Condom-use

About two-fifth (43%) of the young never-married males in Nigeria were sexually experienced, the level of condom-use among them was low—an indication of a high level of their vulnerability to STIs, including HIV. Seventeen percent of the young never-married males who were sexually experienced used condom at the time of their first sexual act (Table 4). Table 4 shows the percentage distribution of respondents who used a condom at their first sexual experience according to selected background factors. Table 4 indicates that condom-use at first sexual initiation varied considerably according to the personal attributes of the respondents, particularly age, urban-rural residence, and region. As would be expected, the proportions of the respondents who reported condom-use at first sexual intercourse increased with age. For instance, 11% of the respondents aged 15-19 years reported condom-use at first sexual intercourse compared to 20% of those aged 20-24 years. The result indicates that condom-use at first sexual initiation tended to be higher among urban dwellers than rural dwellers. About 3 in 10 urban dwellers included in this analysis reported condom-use at the first sexual intercourse compared to 11% for those living in rural areas.

**Table 4.** Percentage distribution of young never-married males, aged 15-24 years, who used condoms during first and last sexual encounters by selected characteristics.

Characteristics/variable	Use at first sex (n=355)		Use during last sex (n=355)	
	No.	%	No.	%
Age-group (years)				
15-19	126	10.6	90	30.8
20-24	278	19.9	236	40.4
Place of residence				
Urban	179	27.3	144	54.1
Rural	225	11.1	182	27.6
Region of residence				
North-central	86	12.4	69	38.5
Northeast	59	8.2	47	18.1
Northwest	51	6.7	47	5.3
Southeast	55	28.0	39	70.8
South-South	71	11.5	55	42.0
Southwest	82	40.0	69	63.1
Childhood place of residence				
City	65	29.2	57	54.3
Town	136	23.9	110	43.9
Countryside	202	8.3	158	27.5
Educational level				
No education	32	0.0	27	0.0
Primary	89	8.8	70	18.1
Secondary	255	17.0	205	43.0
Tertiary	28	63.7	24	86.6
Religious affiliation				
Catholic	87	22.2	68	46.3
Protestant	64	14.8	52	40.2
Other Christians	99	16.9	75	49.8
Muslim	150	15.8	128	27.0
Traditional/others	4	0.0	3	0.0
Wealth quintile				
First	83	7.8	67	14.8
Second	54	8.4	50	24.1
Third	69	10.6	51	28.4
Fourth	89	18.3	70	46.7
Fifth	109	33.2	88	65.2
Frequency of reading newspapers or magazines				
Never	175	7.9	137	20.4
Less than once a week	111	17.2	91	36.9
At least once a week	82	19.0	68	54.1
Almost every day	35	53.6	29	73.6
Frequency of watching television				
Never	85	6.2	71	7.9
Less than once a week	102	14.0	84	37.5
At least once a week	89	12.3	69	54.1
Almost every day	127	29.0	101	57.3
Frequency of listening to radio				
Never	18	5.9	16	13.0
Less than once a week	36	18.9	26	49.9
At least once a week	82	15.1	64	24.8
Almost every day	267	18.0	219	41.2

Source of data: Nigeria demographic and health survey 2003 (24)

Table 4 reveals a large regional difference in condom-use at the first sexual intercourse among young never-married males in Nigeria. The table shows that more young never-married male res-pondents in the southern region used condom at sexual debut compared to those in the north—two-fifths of the respondents in the southwest region used a condom at the first sexual intercourse compared to 8.2% among the respondents in the northeast region. The finding showed that childhood place of residence confirmed the notion of higher condom-use among those who had some urban contact where access to reproductive health services were more than their rural counterparts. Condom-use at the first sexual intercourse was associated with higher levels of education, better access to mass media (newspapers, television, and radio), and higher socioeconomic status as reflected in the wealth quintile (Table 4). The results of the multivariate analysis of the determinants of condom-use at sexual debut are shown in Table 5. The analysis for this behaviour was restricted to those who reported that they have had sex. An odds ratio of greater than one for a particular variable indicates that the respondents in that category were more likely to have had used a condom than were the respondents in the reference or excluded group of the correlate. Odds ratio of less than one implied that the respondents in that category were less likely to have had used a condom than were the respondents in the reference category, and odds ratios of one implied no difference in the likelihood of condom-use compared to the reference group.

**Table 5.** Logit regression results for correlates of young never-married males aged 15-24 years—reports of condom-use at the first and last sexual intercourse, Nigeria, 2003

Characteristics	Condom-use at first intercourse		Condom-use at last intercourse	
	Odds ratio	Standard error	Odds ratio	Standard error
Age-group (years)				
15-19	0.61	0.26	0.67	0.23
20-24	1.00		1.00	
Place of residence				
Urban			1.40	0.52
Rural			1.00	
Region of residence				
North-central	1.00		1.00	
Northeast	1.32	1.14	0.39	0.23
Northwest	1.14	0.96	0.19	0.18
Southeast	1.96	1.58	1.97	1.07
South-South	1.67	1.34	0.89	0.44
Southwest	6.00**	3.90	2.58*	1.26
Educational level				
Below secondary	1.00		1.00	
Secondary	1.98	1.10	2.82*	1.25
Post-secondary	7.49**	5.88	8.88**	6.67
Religious affiliation				
Catholic	1.18	0.80	0.69	0.42
Protestant	0.77	0.46	1.04	0.55
Other Christians	0.80	0.48	1.07	0.52
Muslim	1.00		1.00	
Wealth quintile				
First	1.00		1.00	
Second	0.70	0.65	3.13*	1.74
Third	0.60	0.51	2.61	1.44
Fourth	0.82	0.68	3.67*	2.39
Fifth	0.69	0.64	2.70	1.90
Frequency of reading newspapers or magazines				
Not at all	1.00		1.00	
Less than once a week	1.67	0.80	0.89	0.35
At least once a week	1.36	0.73	1.98	0.93
Almost every day	5.46**	3.63	3.73	2.73
Frequency of watching television				
Not at all	1.00		1.00	
Less than once a week	1.74	1.05	5.04**	2.68
At least once a week	1.17	0.92	2.90	1.96
Almost every day	1.62	1.25	2.13	1.45
Frequency of listening to radio				
Not at all	1.00		1.00	
Less than once a week	7.74*	8.21	6.22	7.42
At least once a week	3.62	3.70	0.88	1.07
Almost every day	2.17	2.20	1.92	2.20
Childhood place of residence				
City	2.32	1.31		
Town	2.37*	1.06		
Countryside	1.00			
Relationship of the last sex partners				
Causal friend/sex workers			6.36**	2.88
Spouse or girlfriend/fiancée		1.00		

Source of data: Nigeria demographic and health survey 2003 (24).

*p<0.05;

**p<0.01;

1.00=Reference category

The findings in Table 5 also reveal that region of residence, wealth quintile, and exposure to television and radio were the important determinants of premarital sex and emerged as the significant determinants of condom-use at the first sexual encounter. Among these factors were region of residence and frequency of listening to radio. Young never-married males living in the southwest part of Nigeria were six times more likely to have used a condom during their first sexual encounter than those residing in the north-central region. The odds of using a condom at the first sexual encounter were associated with frequency of listening to radio. For example, young never-married males who reported listening to radio at least once a week were almost eight times more likely to have used a condom at their first sexual encounter than their counterparts who had never listened to radio. Education was significantly associated with condom-use at the first sexual encounter among the Nigerian young never-married males. Young never-married males with post-secondary education were 7.5% times more likely to have used a condom compared to their reference group (no education) during their first sexual encounter.

About two-fifths (38%) of sexually-active young never-married males used a condom at the time of their last intercourse. Despite a high level of sexual activity among the young never-married males, the level of condom-use among them was low, an indication of high level of their vulnerability to STIs, including HIV. The results in Table 4 further reveal that condom-use during the most recent sexual encounter was higher among young never-married males who were educated, urban dweller, Christian, had urban exposure at childhood, those at the upper end of age-group (among young never-married males), high household socioeconomic status, and frequency of exposure to newspaper, television, and radio. The logistic regression in Table 5 also reveals that region, education, wealth quintile, exposure to television, and relationship of the last sex partners were significantly associated with condom-use at the last sexual encounter among the young never-married males in Nigeria.

## DISCUSSION

This study found a high level of premarital sexual activity among the young never-married males. Age at initiation of sex remained low and current sexual activity high, although the personal and sensitive nature of questions on sexual activity make reports of age and level prone to deliberate misinformation. The results also suggest that a large proportion of young never-married males engage themselves in sexual intercourse without a condom, thus increasing their vulnerability to STIs and unwanted pregnancies. For instance, the level of condom-use at sexual debut was 17%, and 38% used a condom during their last sexual act. The low level of condom-use at sexual debut is comparable with earlier analysis in Nigeria (10) and other sub-Saharan African countries (29-30). Results revealed that initiation of sexual activity and condom-use are influenced by arrays of background, social and environmental factors (9,20-22).

The results of the present study revealed that the young never-married males who were at the greatest risk of adverse reproductive health behaviours appeared to be from families of low economic status, those who resided in rural areas, and those who lived in the countryside during their childhood. This could be explained by the fact that the majority of the young never-married males from these backgrounds might have been denied sex education, thus making them ignorant of what they needed to do for protection. In addition, the parents, the family, and the community members expect that social and traditional norms should be adhered to strictly; however, the influence of sociocultural norms are being eroded as a result of poverty and education, thereby often exposing them to risky behaviour. The social and traditional norms which prohibited sexual intercourse before marriage are being eroded due to increased age at first marriage being observed (3) and exposure to mass-media influence (31-32). Frequent exposure to mass media, attaining higher levels of education, and growing older appeared to be protective factors against unprotected sex, although the young males with these characteristics were more sexually experienced.

The regional patterns of sexual behaviour (ever had sexual intercourse and used a condom) found in this study suggest that ethnicity may be an important factor to be considered when studying adolescents or young people's sexuality and sexual behaviour. However, this influence of cultural norms may be changing due to globalization and education, especially in cities where people are more likely to abandon traditions because of the heterogeneity of populations. Still for many Nigerians, ethnic affiliation, which is usually a regional element, means shared language and norms governing behaviour and sexuality issues. The results of this study support the view that condom-use and age at sexual debut in Nigeria are a function of varied sociocultural factors, including religion, level of exposure to formal education, and urban-rural residence among others.

Apart from the regional differences in sexual behaviour observed in the study, several results showedthe association between the characteristics of youngnever-married males and condom-use. Comparedto rural dwellers, those residing within urban environments were more sexually experienced and more likely to use a condom at the first coitus. Thehigh level of sexual activity among the urban young never-married males can be seen in the context of a permissive environment which favours theattraction and interaction of youths of opposite sex and which, therefore, exposes them to sexual contact. The same factor, however, serves as a protective mechanism by encouraging the use of a condom due to high level of awareness on the intrinsic worth of condom-use. The result of the study also indicates that growing older is associated with sexuality and condom-use among young never-married males in Nigeria. The finding of the study supports the view that sexual activity and condom-use are functions of household socioeconomic status (wealth quintile).

The results of the study shed further light with regard to the challenges facing programmes aimed at improving the reproductive health situation of adolescents and young never-married males in Nigeria and opportunities for intervention. One major challenge is the sizeable number and diverse nature of factors that appeared to influence behaviour of young never-married males. Some of these were particular characteristics of the environment in which they are reared. Therefore, a single, easy-to-implement intervention is unlikely to provide a solution to the high rate of sexual activity and its consequences of teenage pregnancy and vulnerability to STI and HIV/AIDS transmission in Nigeria. This is consistent with the findings of a previous study which revealed that, when in a given setting and a multitude of correlates exists, each having a small impact on sexual behaviour rather than a few correlates, each having a large impact, a single ‘magic bullet’ is unlikely to be found to change sexual behaviour of adolescents significantly (33).

### Conclusions

The results of the study have important policy implications. Since the factors associated with sexual behaviour and condom-use among never-married young Nigerian males vary according to place of residence, region of residence, exposure to mass media, and wealth quintile, programmes may need to position intervention differently for different target populations. The study established that there is a high level of unprotected premarital sex, thus making the population under study vulnerable to HIV infection. In addition, an unwanted pregnancy could lead to termination of education; as a result, young women and men could face a bleak economic and social future. Therefore, behaviour-change communication should be streng-thened with extensive education on sexual abstinence and safe sexual behaviour through culturally-appropriate messages as is currently undertaken by various non-governmental organizations in the country. In particular, more efforts should be made to reach young never-married males in the countryside, who typically had earlier sexual debut and also tended to be more sexually active, with adequate information on behaviour change, prevention of unwanted pregnancies, and protection from STIs, including HIV/AIDS.

## ACKNOWLEDGEMENTS

The authors acknowledge the helpful comments of Dr. Bamikale Feyisetan of the Academy of Education Development, USA, in the preparation of this article. In addition, the authors thank the Marco International and National Population Commission, Nigeria, for making the data of the Nigeria Demographic and Health Survey available for use.
